# Metabolic and Clinical–Nutritional Correlation Patterns in Relation to 3-Year Disease-Free Survival in Locally Advanced Head and Neck Squamous Cell Carcinoma: A Preliminary Analysis

**DOI:** 10.3390/ijms27156715

**Published:** 2026-07-27

**Authors:** Łukasz Boguszewicz, Agata Hajduk-Bieleń, Mateusz Ciszek, Agnieszka Skorupa, Jolanta Mrochem-Kwarciak, Krzysztof Składowski, Maria Sokół

**Affiliations:** 1 Department of Medical Physics, Maria Sklodowska-Curie National Research Institute of Oncology, Gliwice Branch, 44-102 Gliwice, Poland; mateusz.ciszek@gliwice.nio.gov.pl (M.C.); agnieszka.skorupa@gliwice.nio.gov.pl (A.S.); maria.sokol@gliwice.nio.gov.pl (M.S.); 2 1st Radiation and Clinical Oncology Department, Maria Sklodowska-Curie National Research Institute of Oncology, Gliwice Branch, 44-102 Gliwice, Poland; krzysztof.skladowski@gliwice.nio.gov.pl; 3 Analytics and Clinical Biochemistry Department, Maria Sklodowska-Curie National Research Institute of Oncology, Gliwice Branch, 44-102 Gliwice, Poland; jolanta.mrochem-kwarciak@gliwice.nio.gov.pl

**Keywords:** NMR metabolomics, head and neck cancer, disease-free survival, personalized medicine, metabolomics

## Abstract

This retrospective study characterizes the complex associations between serum 1H-NMR metabolomics, clinical nutritional status, and laboratory blood parameters in men with locally advanced head and neck squamous cell carcinoma (LA-HNSCC) in relation to 3-year disease-free survival (DFS). A total of 536 serum samples from 67 patients, collected weekly during radiotherapy or chemoradiotherapy, were analyzed to characterize the metabolic-nutritional landscape. The patients with 3-year DFS exhibited stronger biological links between elevated levels of serum lipids, phosphocholine, branched-chain amino acids (BCAAs), and clinical markers such as BMI, albumin, and prealbumin compared to those with disease recurrence. Furthermore, our findings highlight a novel pattern of disrupted correlations (metabolic decoupling) between body fat distribution, overall obesity, and lipid metabolism, as well as the links between BCAAs and nutritional status, in patients with disease recurrence. This observed breakdown of internal coordination suggests that the survival advantage associated with body fat arises not just from adiposity, but from the body’s ability to metabolically coordinate fat and amino acid turnover with overall energy demands—a trait known as metabolic flexibility. In conclusion, we hypothesize that disease recurrence in men with LA-HNSCC is driven by failure of this system, manifested as a breakdown in the coordination between metabolic and nutritional profiles. The preserved metabolic-nutritional axis between fat turnover and nutrient availability is associated with 3-year disease-free survival in this patient group, although more research is required to fully elucidate the underlying mechanisms.

## 1. Introduction

Squamous cell carcinoma of the head and neck (HNSCC) is the sixth most common cancer worldwide, with a significant proportion of patients (~60%) presenting locally advanced disease (LA-HNSCC) at diagnosis [1,2]. Despite advances in multimodal therapy including radiotherapy, chemoradiotherapy, and induction chemotherapy, the 5-year survival rate remains below 50% [2]. This underscores the critical need to better understand the underlying biological heterogeneity that leads to disparate outcomes in patients with similar clinical profiles [3,4].

Comorbidities, higher clinical stage (TNM), HPV (human papilloma virus) negativity, and age are known factors associated with a higher risk of relapse. The Charlson comorbidity index (CCI) is a proven estimate of overall survival [5,6] and tolerability of treatment [6] in HNSCC. However, some patients show unexpectedly poor or favorable outcomes despite similar clinical profiles, underscoring the need for integrative biomarkers beyond classical indices. Therefore, there is a need for complementary to or independent of CCI biomarkers or models. Molecular information is not directly incorporated into the CCI, but is becoming an important area of multivariate research in HNSCC. Future studies should integrate comorbidity data (e.g., CCI) with molecular profiling to characterize their dynamic interplay during therapy and inform personalized treatment strategies. The following therapeutic approaches are currently being intensively developed in HNSCC treatment: novel treatment strategies that include chemotherapy, molecularly targeted therapy, and immunotherapy [7,8], as well as personalized treatment based on prediction of short-term treatment outcome or survival (overall or over a specified period of years). Prediction of short-term treatment outcomes appears to be the most important in induction chemotherapy (iCHT). iCHT is sometimes used to debulk tumors prior to definitive therapy; however, its benefit remains controversial due to toxicity [9], variable effectiveness [10], and the absence of robust selection criteria [11]. The general or long-term survival prediction is of particular interest in definitive or radical treatment. Recent translational research has explored radiomics [8], genomics [8], clinical data [12,13], and metabolomics [14] to improve the prediction of therapeutic outcomes in HNSCC. Among various translational approaches, metabolomics—particularly serum-based nuclear magnetic resonance (NMR) profiling—offers a non-invasive and reproducible means to capture the complexity of tumor–host metabolic interactions. While recent research has explored radiomics, genomics, and clinical data to understand treatment response, the integration of metabolomic shifts with clinical nutritional status remains underexplored. To our knowledge, this is the first study examining the combined landscape of NMR-derived metabolites and clinical nutritional markers to evaluate biological trajectories in LA-HNSCC.

The most common secondary toxicity of LA-HNSCC treatment is significant weight loss [15]. A higher BMI (being overweight or obese) at the time of diagnosis and/or before treatment is associated with a better outcome [13,16,17,18]. On the other hand, overweight and obesity affect lipid metabolism, which in turn plays a critical role in carcinogenesis, progression of HNSCC [19], and response to HNSCC treatment. The counterintuitive survival advantage of higher BMI, often termed the ‘obesity paradox,’ suggests a complex role of energy reserves and metabolic coordination in treatment response. As revealed in our studies, the iCHT-induced change in blood serum lipids is correlated with primary tumor regression in male patients after iCHT [20]. Furthermore, male iCHT responders can be distinguished from nonresponders based on the pretreatment levels of blood serum lipids, amino acids involved in protein synthesis and degradation, ketone bodies, formate, and N-acetylcysteine [20]. These results are supported by genetic studies, in which genes related to lipid metabolism have been used successfully to predict response to systemic therapy [21] and overall survival [22]. Furthermore, the serum lipid profile may have a prognostic value for overall survival in HNSCC [23,24,25].

This study is a follow-up of our previous work [20,26] and aims to characterize the associations between serum 1H-NMR metabolomics, clinical nutritional status, and laboratory blood parameters in male patients with LA-HNSCC. Our primary objective is to investigate the interaction between these diverse data sets in relation to 3-year disease-free survival (DFS) and to identify the biological patterns, such as metabolic flexibility, that differentiate survivors from patients with disease recurrence. By examining the coordination between metabolic and clinical profiles, we aim to provide preliminary insights into the biological mechanisms associated with treatment outcomes in this patient group.

## 2. Results

### 2.1. The Study Group

The study cohort comprised 67 male patients with LA-HNSCC, among whom 35 (52%) achieved 3-year disease-free survival (Recovery group), while 32 experienced treatment failure, including recurrence, relapse, metastasis, or death (Recurrence group). Disease-free survival (DFS) was defined as the duration following the completion of primary cancer treatment during which the patient remained free of any detectable cancer symptoms. Although both 3-year and 5-year disease-free survival (DFS) profiles were analyzed, the predictive models focus primarily on the 3-year timeframe, where metabolic and clinical divergences were most prominently sustained. The comprehensive validation data and survival curves for the 5-year DFS analysis are provided in the Supplementary Materials (Supplementary Figure S3).

Detailed characteristics of the Recovery and Recurrence groups are presented in Table 1. Statistical evaluation using Mann–Whitney U and χ^2^ tests did not reveal any significant baseline differences in clinical parameters between the groups. However, the patients in the Recurrence group tended to exhibit higher CCI values—a validated estimate of overall survival and treatment tolerability in HNSCC [5,6]. Furthermore, a lower proportion of patients in the Recurrence group received the most intensive treatment modality, induction chemotherapy combined with concurrent chemoradiotherapy (iCHT + cCHRT), often due to the high toxicity profile associated with this approach. The distribution of CCI values and treatment modalities across both groups is further illustrated in the Supplementary Materials (Figure S2).

### 2.2. Identification of Metabolic and Clinical Features Associated with the Disease-Free Survival

Multivariate OPLS (Orthogonal Projections to Latent Structures) modeling was employed to visualize and identify the biological variables that differentiate the Recovery and Recurrence groups. The model analyzed the relationship between two X variables—the time from the start of treatment (days, as a continuous variable) and the group membership (0–1: Recovery vs. Recurrence)—and a set of Y variables consisting of blood serum metabolites, laboratory parameters, and clinical markers measured at weekly intervals throughout the cCHRT/RT course. The fitting parameters of the OPLS model are provided in Supplementary Material (Table S2). The analysis identified a specific metabolic-clinical signature associated with 3-year DFS. This signature included specific 1H-NMR signals: lipids (at 0.9, 1.3, and 5.3 ppm), phosphocholine (Pcho), and branched-chain amino acids (BCAAs: leucine and valine), along with the clinical markers such as BMI, albumin (ALB), and prealbumin (PreALB). The model was validated using a patient-level cross-validation scheme. Under this strategy, the cross-validation groups were strictly blocked on a per-patient basis, which means that all longitudinal measurements from a single patient were entirely excluded together in each validation cycle, eliminating any risk of data leakage between the training and validation steps.

**Figure 1:**
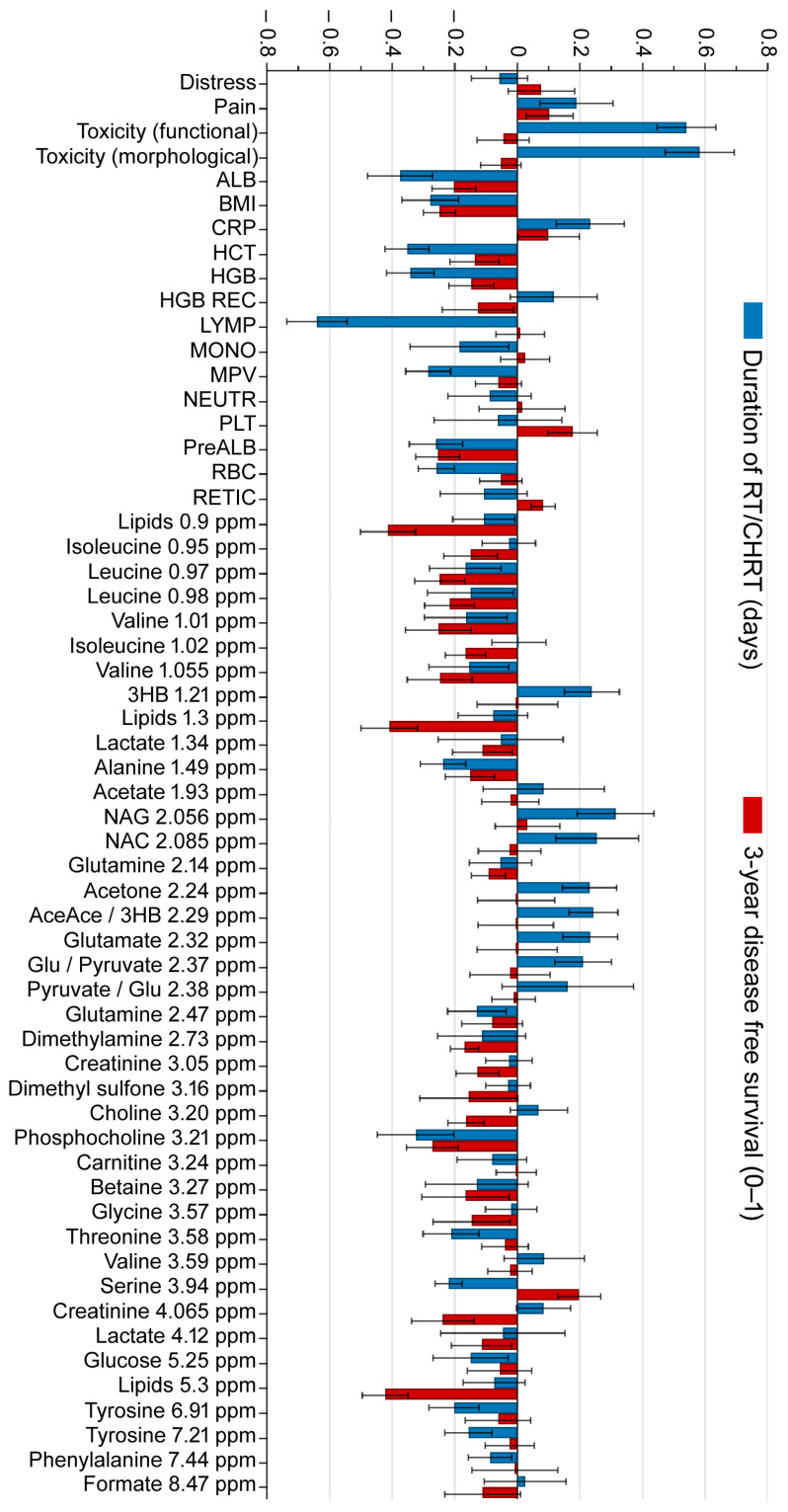
The OPLS coefficient overview plot showing how the 3-year response to the treatment (red bars) and the duration of cCHRT/RT (blue bars) affect the blood NMR metabolites, the blood laboratory parameters, and the clinical parameters. The coefficients are normalized to the range [−1, 1] and are displayed as vertical bars. The height of the bar determines how strongly the analyzed property variable (Recovery/Recurrence, duration of cCHRT/RT) affects a given descriptive variable (blood serum metabolite, blood laboratory parameter, clinical parameter), and the positive and negative values determine the increase or decrease in the value of a given descriptive variable under the influence of the analyzed property variable. The error whiskers represent a 95% confidence interval (derived from jack-knifing). OPLS coefficients are not the variables from the dataset and are completely independent of the type of probability distribution for a given variable—they are presented in the default way for this type of analysis (as normalized bars with confidence intervals). The results take into account the resonance signals of the metabolites at different chemical shifts, e.g., isoleucine at 0.95 and 1.02 ppm. Legend: HCT—hematocrit; ALB—albumin; CRP—C-reactive protein; LYMP—lymphocytes; NEUTR—neutrophil; MONO—monocyte; RBC—red blood cells; HGB—hemoglobin; RETIC—reticulocytes; HGB REC—hemoglobin in reticulocytes; PLT—platelet; MPV—mean platelet volume; PreALB—prealbumin; 3HB—3-hydroxybutyrate; NAG—N-acetyl-glycoprotein; AceAce—acetoacetate; Glu—glutamate; NAC—N-acetylcysteine.

Figure 1 shows the OPLS coefficient overview plot, namely how the X variables affect the Y variables. Positive/negative value indicates the increase/decrease in a metabolite level, respectively, and the error bars represent a 95% confidence interval. The X variables affected the Y variables differently. For example, although lymphocytes decreased dramatically due to their known extreme radiosensitivity (which is reflected in the large blue bar corresponding to the duration of cCHRT/RT), they did not correlate with treatment failure. In contrast, the lipid signals at 0.9, 1.3, and 5.3 ppm showed a robust decrease specifically correlated with recurrence (marked in red), while their change in response to the treatment itself was negligible (Figure 1).

**Figure 2:**
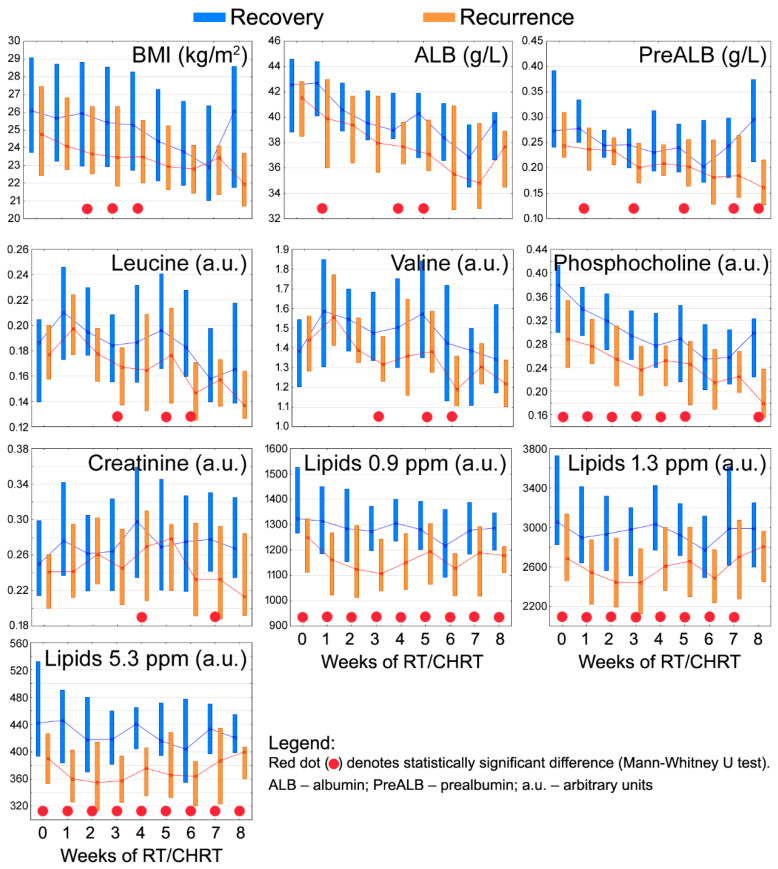
The box plots represent the relative changes during the course of cCHRT/RT in the serum NMR metabolites, the laboratory parameters of the blood, and the clinical parameters identified by OPLS as correlated with 3-year disease-free survival. The lines connect the median points, and the boxes represent the 25–75% percentiles. Differences between the groups were assessed using the Mann–Whitney U test, and the statistically significant time points are indicated with a red dot (●). The relative concentrations of the NMR metabolites are provided in arbitrary units (a.u.). Although these values reflect physiological levels, they are not identical to absolute concentrations. Nevertheless, this format is highly suitable for robust inter-group comparisons.

The variables with an OPLS coefficient > 0.2 were selected for further longitudinal examination (Figure 2). To evaluate the temporal dynamics of individual metabolites and clinical parameters, group differences (Recovery vs. Recurrence) were assessed cross-sectionally at each individual time point using independent Mann–Whitney U tests. Because each patient contributed exactly one sample per weekly analysis, the assumption of independent observations within each test was preserved. The lines in Figure 2 connect the median points, and the boxes represent the 25–75% percentiles. The differences between the Recovery and Recurrence groups were evaluated using the MWU test, and the statistically significant time points are indicated with a red dot (●). Patients in the Recovery group exhibited significantly higher serum lipid and phosphocholine levels throughout most of the cCHRT/RT course compared to those in the Recurrence group. Furthermore, significantly higher values for BMI, ALB, and PreALB, as well as leucine and valine, were observed in the Recovery group, with the statistical significance peaking primarily during the midcourse of treatment.

### 2.3. Validation of the Biological Signature Using an External Test Set

To evaluate the robustness and consistency of the identified metabolic-clinical signature using an independent external test set, while simultaneously leveraging the comprehensive multi-week monitoring protocol and preserving statistical independence, the final predictive framework was transitioned to a hierarchical batch-modeling workflow (BEM and BLM). This model, trained strictly on the primary cohort (described in detail in Section 4.5 of Materials and Methods) was limited to the ten prioritized X variables (BMI, PreALB, and NMR signals of leucine, valine, phosphocholine, and lipids at 0.9, 1.3, and 5.3 ppm) and DFS as the binary Y variable. The fitting parameters of the BEM and BLMs are provided in Supplementary Material (Table S2).

The predictive power of this batch-level signature was evaluated using an independent external test set of 36 HNSCC patients (19 with 3-year DFS and 17 with treatment failure). Detailed characteristics of the external test set are provided in the Supplementary Material (Table S3). These individuals were deliberately excluded from the main training cohort to maintain its homogeneity with respect to HPV status (*n* = 10 unknown, *n* = 13 positive) and treatment modality (*n* = 13 were treated with RT alone). Of 13 patients treated with RT, 12 were HPV negative, one more HPV patient received induction chemotherapy followed by cCHRT. A total of 266 longitudinal NMR samples were analyzed. Crucially, the test set samples were not involved in any data preprocessing steps used during the initial development of the prediction model, ensuring a truly independent assessment.

**Figure 3:**
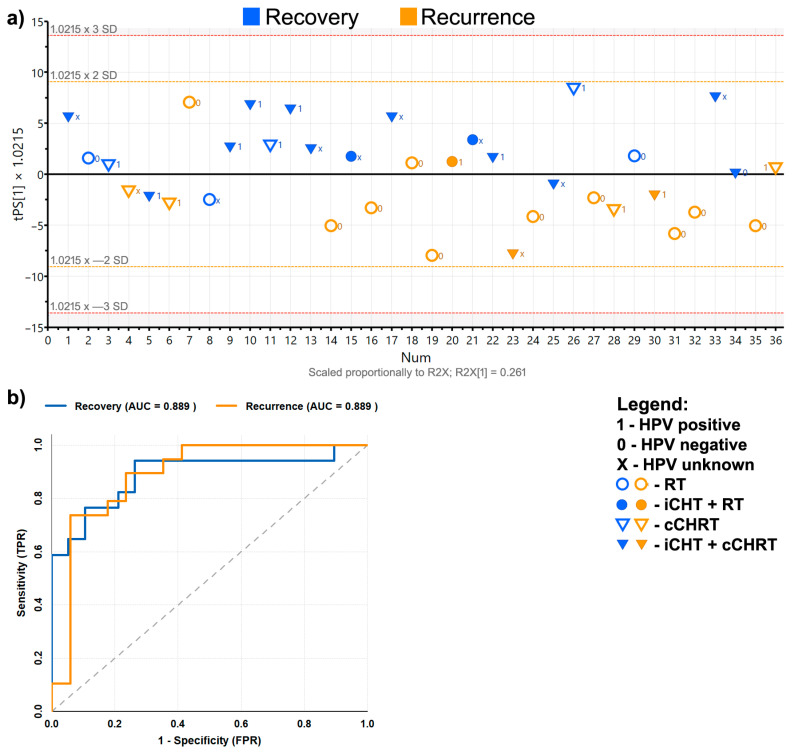
The results of the 3-year DFS prediction for the external test set. The data from 36 patients were used for prediction. The plot of the predicted scores (tPS) (**a**) shows the results obtained by the OPLS-DA batch-level model constructed for the study group. The receiver operating curve (**b**) shows a very high predictive capacity of the model with an area under the true positive rate (TPR) curve of 0.889. The significance of the observed classification was evaluated with Fisher’s probability test with the significance threshold of 0.05.

The longitudinal profiles of these test subjects were blindly projected into the frozen-training BLM space. The result of this prediction is visualized in Figure 3. The predicted scores plot (Figure 3a) demonstrated a robust capacity to assign unknown patient trajectories to the correct clinical category, showing a clear and distinct separation between the Recovery and Recurrence groups along the predictive axis.

To quantitatively evaluate this classification, continuous predictive values (Y-pred) representing the classification probability for each patient were extracted using the Y-Predicted List tool in SIMCA software (Sartorius Stedim Data Analytics, v. 18, Umeå, Sweden). Using a standard decision threshold of 0.5, patients were assigned to predicted clinical categories. The significance of this external classification was confirmed using Fisher’s exact test (*p* < 0.0002). Furthermore, a receiver operating characteristic (ROC) analysis based on these continuous Y-pred probabilities yielded an area under the curve (AUC) of 0.889 (Figure 3b), with the true positive rate confirming high consistency between both groups. These results, although preliminary given the size of the cohort, suggest that the associations identified between specific metabolites and nutritional markers represent a distinct biological pattern that correlates with clinical outcomes in this group of patients with LA-HNSCC.

### 2.4. Disruption of Metabolic-Nutritional Interplay

Serum lipids and branched-chain amino acids (BCAAs) are physiologically associated with nutritional status, adiposity, and metabolic health, including conditions such as overweight, obesity, and diabetes. Our analysis examined how these established biological links manifest themselves in the patients with LA-HNSCC in relation to clinical outcomes.

In the Recovery group, a well-integrated metabolic-nutritional network was clearly observed, characterized by significant positive correlations between serum lipids, BCAAs, and clinical markers of nutritional status, including BMI, ALB, and PreALB (Table 2). In contrast, these fundamental associations were largely disrupted in the Recurrence group, where the correlations between the lipids, BCAAs, and nutritional indices were mainly statistically insignificant. The results presented in Table 2 show the values of Spearman correlation (R) parameters between the NMR signals of lipids and BCAAs and the clinical nutritional parameters; the statistical significance (*p*-value < 0.05 and R > 0.3) values are denoted with a bold font.

Thus, we hypothesize that this breakdown of internal coordination indicates that treatment failure is not only associated with lower metabolite levels, but with a fundamental loss of metabolic-nutritional integration—a hallmark of impaired metabolic flexibility. This distinctive loss of coordination between the body’s metabolic profile and its nutritional reserves appears to be a key feature of disease recurrence in this patient group.

### 2.5. Assessment of Factors Influencing the Biological Interpretation of the Results

To ensure a robust biological interpretation of the observed patterns, the internal correlation structure of the clinical parameters was evaluated to identify potential confounding factors (Table 3). Table 3 presents the Spearman correlation coefficients between the selected parameters; the significant values (with *p* < 0.05) are bolded. One of the factors used to calculate CCI is the patient’s age, hence the very strong correlation between these two variables. Overall, the observed differences in the correlation patterns between the two Recovery and Recurrence groups are minor and limited to two main areas. In the Recovery group, tumor grade showed strong correlations with nodal stage (N) and clinical stage (TNM), associations that were notably absent in the Recurrence group. In the Recurrence group, the modality of treatment (iCHT vs. no iCHT) was significantly correlated with age and CCI, reflecting the clinical tendency to omit intensive induction chemotherapy in older patients with higher comorbidity.

**Figure 4:**
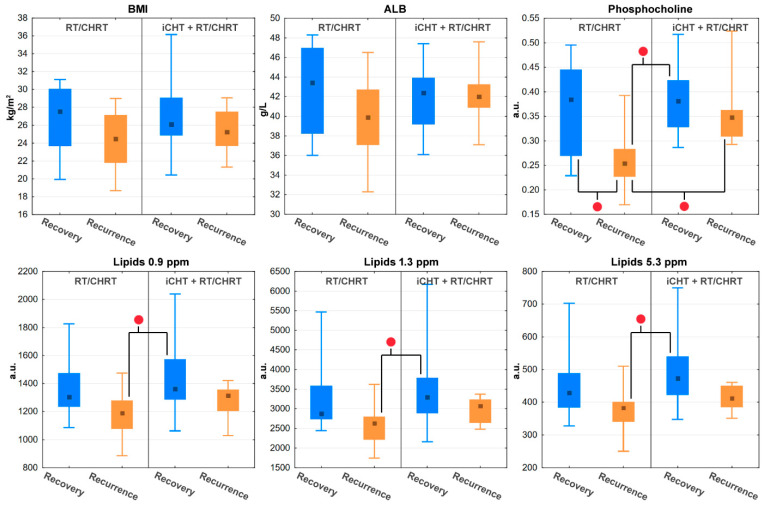
The differences in the baseline (the day 0 of the multiparametric monitoring—the day RT/CHRT treatment starts) body mass index (BMI), albumin (ALB), phosphocholine, and blood serum lipids between Recovery and Recurrence groups and between the patients who were/were not treated with iCHT. The boxes represent the 25–75% percentiles, the points within them are the median values, and the whiskers denote the minimum and maximum values. A statistical significance (red dot, ●) was assessed with Kruskal–Wallis ANOVA (KWA). The number of included samples is: RT/CHRT Recovery, *n* = 14; RT/CHRT Recurrence, *n* = 19; iCHT + RT/CHRT Recovery, *n* = 21; iCHT + RT/CHRT, *n* = 13; and corresponds to the data provided in Table 1. a.u.—arbitrary units.

As our previous studies have shown [20], induction chemotherapy (iCHT) leads to a significant increase in serum levels of lipids, phosphocholine, and albumin. Given that iCHT was used less frequently in the Recurrence group (the difference-approaching statistical significance), it was crucial to determine whether these variables independently distinguish clinical outcomes or if the observed differences were simply a side effect of the treatment modality. To address this, we evaluated the baseline distribution (at Day 0 of the multiparametric monitoring) of BMI, ALB, Pcho, and lipids (0.9, 1.3, 5.3 ppm) in four subgroups: Recovery and Recurrence, each stratified by the presence or absence of prior iCHT (Figure 4). Since this analysis evaluated the metabolic effects at Day 0 (prior to the commencement of the next treatment phase), subsequent treatment modalities—such as RT or cCHRT—had not yet exerted any biological effect. Consequently, the subgroups sharing the same induction history were combined to robustly assess the impact of induction chemotherapy. The number of included samples is: RT/CHRT Recovery, *n* = 14; RT/CHRT Recurrence, *n* = 19; iCHT + RT/CHRT Recovery, *n* = 21; iCHT + RT/CHRT, *n* = 13; and corresponds to the data provided in Table 1. Statistical significance was assessed using Kruskal–Wallis ANOVA (KWA).

The analysis confirmed that while iCHT indeed shifts the metabolic profile towards higher levels of lipids and Pcho, it does not mask the underlying biological differences between the clinical groups. A clear overall trend was observed, with the Recurrence group consistently showing lower-baseline lipid levels compared to the Recovery group, regardless of the treatment history (Figure 4). However, statistical significance (KWA) was specifically reached between the Recurrence patients who did not receive iCHT and the Recovery group who did receive iCHT. Furthermore, the Recurrence (no iCHT) subgroup showed significantly lower-baseline phosphocholine levels than the entire iCHT-treated cohort (Figure 4). These findings demonstrate that despite the modulating effect of iCHT, the metabolic-nutritional disparity between survivors and those with treatment failure remains evident, pointing toward an intrinsic biological divergence.

## 3. Discussion

### 3.1. Prognostic Performance and Rationale for Multivariate Pattern Recognition

This retrospective study combined NMR-based metabolomics with clinical nutritional markers (BMI, albumin, prealbumin) to characterize the biological landscape and metabolic-nutritional interactions associated with disease-free survival (DFS) in patients with locally advanced head and neck squamous cell carcinoma (LA-HNSCC). While the core predictive models focus on the 3-year DFS timeframe—which offers a clinically actionable window for intervention—the comprehensive analyses and longitudinal validation data for the 5-year DFS are provided in the Supplementary Materials. Our results were obtained from routinely collected blood samples, making the procedure virtually non-invasive and easy to implement in clinical practice. The potential for real-time DFS prediction during radiotherapy (RT/cCHRT) could offer a way to both assess patient-specific responses and guide adaptive clinical decision-making.

The variables associated with clinical outcome were first identified using a supervised multivariate regression method (OPLS) and subsequently validated using an OPLS-DA classifier. The model achieved high predictive accuracy in distinguishing between the Recovery and Recurrence groups within the external test set for both survival milestones. Specifically, the classifier demonstrated excellent discriminative power, yielding an AUC of 0.889 for the 3-year disease-free survival (DFS) endpoint and an AUC of 0.866 for the 5-year DFS endpoint (Supplementary Material), with Fisher’s exact test confirming statistical significance for both models. Although the current validation involved all available measurements for each patient during the treatment period, the ultimate goal is to apply this model on an ongoing basis to monitor the probability of cure in real time as treatment progresses.

In our analysis, the statistically significant differences for individual parameters (excluding lipids and phosphocholine) were sparse. However, the strength of multivariate analysis lies in its pattern recognition capability. As noted previously [27], multiple variables following a consistent biological trend provide a more robust and reliable result than any single variable with an acceptable *p*-value. Univariate approaches treat variables independently, ignoring correlations between them, which often leads to false positives. A central feature of metabolomic data, in particular spectral data, is its high degree of collinearity. Because metabolites act together in complex metabolic networks, optimal biological insight can only be achieved when using methods that take into account the correlation between metabolites [28]. If multiple variables follow a consistent biological trend, the “pattern” is far more likely to be true biology rather than chance, providing higher confidence in the results, and allowing for the discovery of hidden patterns, control of confounding factors, and the identification of complex biological phenotypes that surpass the limitations of univariate analysis. Although the metabolic trends during RT/cCHRT follow a similar trajectory in both groups, a persistent disparity remains evident (Figure 2), allowing the identification of factors that influence recovery.

### 3.2. Clinical Relevance of Baseline Nutritional Status and the “Obesity Paradox”

One of such critical factors identified in our cohort is nutritional status; the Recovery group exhibited higher values of BMI, ALB, and PreALB. In particular, no patient was underweight (BMI < 18.5) at the beginning of treatment. However, while 62.5% of the Recurrence group fell within the normal BMI range (18.5 < BMI < 24.9), the Recovery group was characterized by a higher prevalence of overweight (50%) (25 < BMI < 29.9) and obesity (17.6%) (BMI > 30). In both studied groups, the albumin values were in the normal range (34–54 g/L) for all patients, while PreALB values were above the normal range (0.16–0.3 g/L) in three and ten patients in the Recurrence and Recovery groups, respectively.

Unlike many other malignancies, such as breast or pancreatic cancer, where obesity worsens the prognosis, HNSCC is characterized by an “obesity paradox” [16]. It means that being overweight at the time of cancer diagnosis is a prognostic factor for better survival [16,17,18]. Furthermore, being overweight or obese has been significantly associated with a reduced risk of developing squamous cell carcinoma of the head and neck [29]. Although exact reasons are still being explored, they likely involve factors such as reduced catabolic state, superior energy reserves to combat treatment-related weight loss, and specific protective effects of adipose tissue [16,18]. However, the exact biological mechanisms behind this paradox are not fully understood and require more molecular studies, particularly with regard to lipid metabolism—as the relationship between body fat distribution, general obesity, and lipid metabolism is well-documented [30,31].

Our study provides a deeper hypothesis-generating mechanistic insight into this paradox by using NMR, which has been shown to be an accurate technique to characterize lipid profiles in such clinical contexts [32,33].

### 3.3. Proposed Biological Framework: Alteration in Lipid, Phosphocholine and BCAA Metabolism

In the Recovery group, the levels of NMR-measured serum lipid signals are significantly higher during cCHRT/RT compared to patients in the Recurrence group. In NMR spectroscopy, the signals at 0.9 ppm, 1.3 ppm, and 5.3 ppm correspond to terminal methyl groups (CH_3_), methylene groups (CH_2_) within saturated fatty acyl chains, and to olefinic protons (-CH=CH-) in unsaturated fatty acids, respectively [20]. While alterations in lipid profiles are evident in HNSCC [34], it is consistently shown that these patients have reduced levels of serum lipids compared to healthy controls [35]. One of the main reasons for this is the immune response to radiotherapy and chemoradiotherapy, which increases systemic low-grade inflammation [36]. In our study, both groups experienced this therapy-induced effect; however, the persistent difference between their lipid profiles suggests a metabolic maladaptation in the Recurrence group. In these patients, the inability to maintain systemic lipid levels despite nutritional reserves points toward a fundamental disruption of the host–tumor metabolic balance, signaling a shift toward a more aggressive disease phenotype [37].

Phosphocholine (Pcho) levels were found to be significantly higher in the Recovery group, although both groups exhibited a gradual decrease in this metabolite during therapy. A more granular analysis of baseline values (Day 0) revealed that patients in the Recurrence group who did not receive induction chemotherapy (no iCHT) started with significantly lower Pcho levels compared to both the Recovery group without iCHT and the entire cohort treated with induction therapy (Figure 4). The persistence of this difference, despite the modulating effects of iCHT, points to intrinsic metabolic resilience in survivors. Phosphocholine serves as the headgroup for phosphatidylcholine (PC), which is the main phospholipid in eukaryotic cell membranes, often making up 30–60% of the total phospholipid mass. Elevated levels of this crucial cell membrane precursor have been consistently reported in most MAS NMR studies of various tumor types. This increase in phosphocholine reflects both cell death (apoptosis and necrosis) and proliferation [38]. Although phosphocholine levels can reflect a shared physiological response to specific therapeutic interventions, the consistent disparity observed here suggests a deeper biological distinction. Higher levels of Pcho in the Recovery group than in the Recurrence group probably reflect a more robust state of membrane turnover and systemic cellular signaling, potentially indicating a superior capacity for tissue repair and regeneration during the physiological stress of radiotherapy [39]. Why this biological advantage remains stable throughout treatment remains a subject for further investigation, but it underscores Pcho as a key component of the host’s metabolic-nutritional integrity.

Other important metabolites that influenced the group separation were branched-chain amino acids (BCAAs), mainly valine and leucine. Beyond their role as protein building blocks, these amino acids are critical for cellular signaling pathways, energy metabolism, and immune regulation, all of which influence tumor progression [40] and undergo distinct, cancer-specific metabolic reprogramming driven by diverse genetic backgrounds of different tumors [40]. In fact, BCAAs play a dual role in malignancies: their high concentrations can support overall metabolic health, but their deficiency promotes metastasis by disrupting tumor cell adhesion and angiogenesis [40,41]. Therefore, the higher levels of BCAAs observed in our Recovery group probably represent a favorable systemic environment that supports treatment tolerance and inhibits aggressive disease progression through preserved metabolic-nutritional coordination.

Recent studies indicate that in head and neck squamous cell carcinomas BCAA metabolism is reprogrammed, typically promoting cancer progression, therapy resistance, and immunosuppression. The exact mechanisms are still being elucidated, but evidence suggests that high levels of BCAA catabolism are associated with poor prognosis and increased tumor aggressiveness. High catabolic demand by tumors can lead to a systemic reduction in BCAAs availability in the blood, a phenomenon often associated with severe cancer cachexia and disease progression. Systemic decline in BCAAs is a key metabolic alteration in head and neck cancer patients that often precedes and contributes to muscle mass loss and cancer cachexia [20,26]. In our current study, the elevated levels of BCAAs in the sera of the Recovery group may be interpreted not only as a correlate of a higher BMI but also as a reflection of superior muscle mass and overall physical condition. This metabolic reserve likely provides a “protective buffer” during the catabolic stress of RT/cCHRT, as higher muscle mass is associated with increased BCAA oxidation during treatment. This is particularly relevant given that sarcopenic HNSCC patients, even those without obesity, have been reported to have poorer overall survival [42]. On the contrary, the significant decline in BCAAs in the sera of the Recurrence group may signify a state of “metabolic exhaustion”, where the inability to maintain BCAA homeostasis becomes a hallmark of biological vulnerability. Although detailed body composition was not measured in this study, the positive correlation between BCAAs and BMI (especially in men) [43], obesity [44], diabetes [45], and muscle preservation is well-documented in non-cancer subjects [46].

### 3.4. Proposed Theoretical Model: Metabolic Flexibility Versus Functional Decoupling

The correlation patterns between serum lipids, BCAAs, and nutritional markers seem to support the above statements revealing distinct profiles between the Recovery and Recurrence groups. The Recovery group shows a strong correlation between the baseline serum lipids and BMI and PreALB, as well as between the baseline BCAAs and BMI and ALB, and all of these correlations persist largely during RT/cCHRT (Table 2). In the Recurrence group, baseline serum lipid levels correlate only with ALB, and this correlation disappears after the first week of RT/cCHRT, and BCAAs do not show a correlation with nutritional markers (Table 2).

In summary, our study adds a crucial, hypothesis-generating mechanistic layer to the understanding of the so-called “obesity paradox” in HNSCC. Although obesity can promote tumor development and progression, obese patients often show a survival advantage compared to their normal weight or underweight counterparts [16,42]. Our results suggest that this advantage may not be derived from adiposity per se, but rather from a functional and preserved metabolic-nutritional axis. The survival benefit likely arises from the body’s ability to metabolically coordinate fat and amino acid metabolism with overall energy demands and nutrient availability—a trait known as metabolic flexibility. Conversely, the breakdown of these correlations in the Recurrence group potentially indicates a functional decoupling, where systemic reserves appear to be no longer integrated with metabolic needs. In this context, we hypothesize that HNSCC does not only affect lipid synthesis and catabolism [35,47,48], but could also lead to a fundamental loss of metabolic coordination, which may represent a hallmark of poor clinical outcome. These findings highlight the biological importance of incorporating metabolic and nutritional profiling into the characterization of LA-HNSCC, offering a deeper understanding of host-specific responses to treatment.

### 3.5. Influence of Clinical Covariates, Treatment Modalities, and Study Limitations

Although only close to statistical significance, the patients in the Recovery and Recurrence group were different in terms of treatment modality and CCI (Table 1). In LA-HNSCC clinical practice, treatment is highly customized according to the patient’s baseline condition. Consequently, in our Recurrence group, where patients have a higher CCI, iCHT is used less frequently (Table 1, Figure S2). Furthermore, within the Recurrence group, ALB was significantly negatively correlated with CCI, while in the Recovery group, CCI was significantly negatively correlated with PreALB, as well as with the valine and isoleucine signals. No correlation of CCI with lipids was observed.

While iCHT results in elevated serum lipids [20], these differences were not statistically significant in our study group, except for the patients in the Recurrence group. They start RT/cCHRT with significantly lower serum lipids than the Recovery patients who received iCHT (Figure 4). Crucially, the persistent metabolic disparity between the groups, regardless of the status of iCHT, suggests that the observed metabolic-nutritional signature reflects an intrinsic biological characteristic of the host rather than being a mere artifact of the treatment modality.

Among the limitations of this study, the relatively small and retrospective study group should be addressed. To mitigate this, we used rigorously collected longitudinal data supplemented with long-term survival information, selecting the largest possible subgroup while maintaining high cohort homogeneity. Another limitation is the exclusive inclusion of male patients. Based on our previous findings, we hypothesize that metabolic profiles and responses to therapy differ between sexes [20]. Our supposition is confirmed by recent literature reports. Biological sex is claimed to be a crucial determinant in head and neck squamous cell carcinoma: men have a 2 to 3-fold higher incidence, yet women often present with biologically favorable antitumor immune landscapes, although they historically experience complex, distinct toxicity, and survival trajectories across varying therapies [49]. Genetic and environmental factors may contribute to these differences and affect cancer patient health outcomes [50,51,52,53]. All these observations indicate that future HNSCC studies require a separate analysis for women and underscore the need for sex-specific medicine.

Furthermore, a notable characteristic of the external test set is its heterogeneous distribution of HPV status, consisting of 13 positive, 13 negative, and 10 unknown cases (Table S3), contrasting with the strictly HPV-negative profiling of the main discovery cohort. At the tissue level, HPV-positive and HPV-negative HNSCC tumors are known to exhibit distinct cell-intrinsic metabolic wirings, with HPV-negative cells often displaying higher glycolytic dependence. However, the highly robust performance of our classifier within this mixed test set (yielding high predictive accuracy for both survival milestones—Figure 3 and Figure S3) demonstrates that the identified prognostic metabolic-nutritional axis operates independently of this molecular stratification. This implies that while tumor-intrinsic metabolomic profiles are heavily shaped by viral etiology, the systemic serum metabolome captured in our study primarily reflects the host’s global metabolic flexibility, nutritional reserves, and systemic capacity to withstand therapeutic stress. Consequently, this systemic integrity is hypothesized to act as a potential prognostic framework across different molecular subtypes of HNSCC, rather than a subtype-restricted phenomenon.

While induction chemotherapy (iCHT) has been previously demonstrated to modulate systemic metabolism and elevate serum lipid levels in HNSCC patients [20], its potential confounding effect on our prognostic model was carefully considered. To address this, we compared the baseline (Day 0) metabolic and nutritional profiles of the Recovery and Recurrence groups stratified by their prior iCHT status (Figure 4). Our sub-analysis reveals that although iCHT is associated with a general upward trend in serum lipids, these intra-group differences did not reach statistical significance; the patients within the Recovery group who received iCHT exhibited lipid profiles statistically comparable to those who underwent RT/cCHRT alone. Crucially, while numerical disparities in the lipid levels between the Recovery and Recurrence groups are visually apparent across both treatment arms, statistically significant inter-group differences emerged primarily when contrasting the high-baseline Recovery patients modulated by prior iCHT against the lower-baseline Recurrence counterparts (Figure 4). This pattern suggests that while iCHT may act as a modulating factor that accentuates these metabolic variances, the underlying directional trends remain consistently preserved. Therefore, our signature is hypothesized to capture an intrinsic biological phenotype that is further highlighted, rather than artificially created, by the selection for induction chemotherapy.

Finally, since the study focused solely on HNSCC, it remains to be determined whether these results are specific to this malignancy or reflect a more general pattern of cancer–host metabolic interactions under therapeutic stress.

### 3.6. Conclusions

In conclusion, our study provides preliminary evidence to hypothesize that 3-year disease-free survival in male patients with LA-HNSCC is associated with a more integrated metabolic-nutritional landscape. The preservation of correlations between serum lipids, BCAAs, and clinical markers (BMI, albumin, prealbumin) may point to metabolic flexibility as a significant potential biological factor in favorable outcomes. On the contrary, the observed breakdown of these associations—suggesting metabolic decoupling—could reflect a systemic failure of the host to coordinate energy reserves with metabolic demands during therapy. These findings offer a hypothetical mechanistic basis for the ‘obesity paradox’ in HNSCC and underscore the possible importance of the metabolic-nutritional axis in maintaining systemic integrity. Although these patterns were observed in a relatively small cohort, the holistic evaluation of these biological trajectories offers deeper insight into the complex host–tumor interaction and highlights the need for further research to evaluate whether preserving metabolic coordination could serve as a potential target for therapeutic support.

## 4. Materials and Methods

### 4.1. Patients

This retrospective study was conducted according to the guidelines of the Declaration of Helsinki and approved by the Institutional Review Board (Ethics Committee) of the Maria Sklodowska-Curie National Research Institute of Oncology (protocol code DO/DGP/493/4/06/1/2016/G and date of approval 26 January 2016) and informed consent was obtained from the participants.

The studied group consisted of 67 Caucasian male patients with HNSCC. The patients were radically treated in the 1st Department of Radiation and Clinical Oncology of the Maria Sklodowska-Curie National Cancer Research Institute, Gliwice Branch, Poland. All were pathologically confirmed (biopsy-proven) with squamous cell carcinoma (according to the third edition of the WHO scale) and with the clinical (TNM) stages III to IVb (according to the seventh edition of the American Joint Committee on Cancer (AJCC)). The patients had a primary tumor of the oropharynx, hypopharynx, nasopharynx, and larynx, and all patients were HPV negative. Inclusion was restricted to male patients to ensure high biological homogeneity, based on our previous research that indicated significant gender-specific molecular differences in the response to treatment [20]. This approach allowed for a more focused characterization of the metabolic-nutritional axis by minimizing gender-related metabolic variability. All participants had a performance status less than 2 (ZUBROD WHO scale), good nutritional status, and were inoperable or suitable for organ preservation strategy.

The exclusion criteria were as follows: distant metastases; squamous cell carcinoma of the nose, sinus, ear, skin, thyroid, or salivary glands; a previous secondary malignancy; a performance status greater than or equal to 2 (ZUBROD WHO scale); or no permission for chemotherapy.

The patients were treated with:

(a) Concurrent chemoradiotherapy, cCHRT, using a conventional fractionation technique (2 Gy per fraction, 35 fractions, total dose 70 Gy, administered once a day and 5 days a week with a weekend break for 7 weeks) and cisplatin fractionated from the first day of radiotherapy (RT), with a 21-day interval (2–3 cycles). *n* = 33 patients.
(b) Modality (a) preceded by induction chemotherapy (iCHT) performed with 3–4 cycles of TPF (the chemotherapy combination that contains the drugs docetaxel (Taxotere), cisplatin (Platinol), and 5-fluorouracil), *n* = 13 or 3 cycles of PF (a combination of cisplatin and 5-fluorouracil), *n* = 9.
(c) RT using continuous accelerated irradiation (1.8 Gy per fraction, 40 fractions, the total dose 72 Gy, administered once a day and 7 days a week, for 6 weeks) preceded by 3–4 cycles of TPF (*n* = 5) or 3 cycles of PF (*n* = 7) iCHT.

The detailed characteristics of the patients are presented in Table 1.

Radiotherapy or concurrent chemoradiotherapy, sometimes preceded by induction chemotherapy, is the treatment of choice for organ preservation in LA-HNSCC. The outcome depends on many factors, the most obvious being the delivery of the planned dose at the planned time, regardless of whether it is radiotherapy or chemotherapy [54]. In the study group, all patients completed treatment as planned.

### 4.2. Study Design

The graphical design of the study is given in the Supplementary Material (Figure S1).

The patients were clinically monitored weekly, from the day before the initiation of concurrent chemoradiotherapy (cCHRT) or radiotherapy (RT) (week 0), through the week after completion of the treatment (week 7), totaling 8 time points per patient. This monitoring included medical, psychological, and endoscopic examinations, laboratory blood tests (evaluating hematologic and nutritional status, renal and liver function, and inflammatory markers), as well as 1H NMR spectroscopy of blood serum [20,26].

Medical and endoscopic evaluations were designed to identify both morphological and functional toxicities associated with treatment. Functional toxicity refers to disturbances in physiological functions such as swallowing difficulties (dysphagia), breathing impairment (dyspnea), hoarseness (dysphonia), taste and smell disturbances (dysgeusia, hyposmia), as well as neuropathic symptoms, including burning and tingling sensations. Morphological toxicity, on the other hand, encompasses visible tissue damage, including acute radiation-induced mucosal reactions (erythema, ulceration, epithelial desquamation), as well as later manifestations, such as fibrosis, glandular atrophy, or osteoradionecrosis, detectable by clinical examination, imaging, or histopathology.

Follow-up ear, nose, and throat (ENT) examinations were performed every six months after treatment, and radiological and laboratory follow-up evaluations were performed annually.

The integrated data were used to characterize the biological trajectories associated with both 3-year and 5-year DFS as exploratory endpoints. We focused our analysis on disease-free survival (DFS) because it offers a robust, unconfounded endpoint that directly reflects the biological propensity for recurrence. Unlike overall survival, DFS is not influenced by salvage treatments or comorbidities that arise after relapse, allowing for a clearer attribution of biological significance to early metabolic and nutritional signatures. Furthermore, DFS precisely targets the clinical need for understanding the biological mechanisms that differentiate long-term survivors from patients predisposed to treatment failure, improving the fundamental insights into metabolic-nutritional coordination in LA-HNSCC.

### 4.3. Sample Collection and Preparation

The peripheral venous blood samples were collected once a week (from week 0 to week 8 for cCHRT and to week 7 for RT) after an overnight fast, corresponding to the differing durations of the conventional versus accelerated radiotherapy schedules. The procedures were performed in a hospital setting. A total of 588 samples were initially collected; however, 52 samples were excluded due to insufficient material (*n* = 48) or low quality of the NMR spectra (*n* = 4). The remaining 536 samples were divided into two aliquots, one of which was used for laboratory blood tests, and the other for 1H NMR spectroscopy. For the NMR analysis, samples were incubated for 30 min at room temperature and then centrifuged (1000× *g*, 10 min) to remove the clot and stored frozen at −80 °C. On the day of measurement, the samples were thawed in two steps (at 4 °C and then at room temperature) and mixed with a phosphate buffer (pH 7.4) containing deuterium oxide (D2O) and trimethylsilylpropionate (TSP). Aliquots of 600 µL of the solution were transferred to 5 mm Wilmad WG-1235-7 NMR tubes (Wilmad Labglass, Vineland, NJ, USA) and maintained at 4 °C until analysis.

### 4.4. NMR Spectroscopy

The same measurement and post-processing protocol based on the protocol proposed by Beckonert et al. [55] was applied as in our previous metabolomic studies [20,26]. The 1H NMR spectra were acquired on a Bruker 400 MHz Avance III spectrometer (Bruker Biospin, Ettlingen, Germany) equipped with a 5 mm PABBI probe. Quality control tests were performed on each measurement day. The detailed measurement and post-processing procedures are described in the Supplementary Materials.

NMR probe tuning and matching, shimming, determination of the transmitter offset value for the water pulse presaturation, and 90° pulse adjustments were performed for each sample. The receiver gain was set to 90.5, and the temperature was maintained at 310 K for all experiments. Four different 1H NMR spectra—NOESY (1D Nuclear Overhauser Enhancement SpectroscopY), CPMG (Carr–Purcell–Meiboom–Gill), diffusion-edited (DIFF), and J-resolved (JRES)—were acquired for each serum sample. The characteristics of the acquired spectra, as well as the pulse sequence parameters, are provided in the Supplementary Materials (Table S1).

To mitigate batch or drift effects, the samples were measured in random order, paying particular attention to avoiding the measurement of multiple samples from the same patient on a single day.

### 4.5. Statistical Analysis

Before starting statistical analyses, the validity of the data was verified, with particular emphasis on outliers, using unsupervised multivariate methods. Four outlying samples were removed.

The main multivariate analyzes were performed on the 536 samples using SIMCA software (Sartorius Stedim Data Analytics, v. 18, Umeå, Sweden). Each sample was described by 58 descriptive variables (40 quantified NMR signals and 18 nutritional, psychological, and standard laboratory blood parameters) and 2 property variables (disease-free survival in 3 years: 0–1 variable; days of cCHRT/RT treatment, a continuous variable). The descriptive variables were scaled to unit variance (UV) and mean-centered prior to modeling. Orthogonal partial least squares analysis (OPLS) was used to identify to what extent specific descriptive variables were associated with the property variables. OPLS combines an orthogonal signal correction with partial least squares analysis (also known as projection to latent structures, PLS); a detailed description of OPLS is available in [56,57]. In summary, orthogonal signal correction removes variation from the descriptive variables (NMR metabolites and clinical parameters) that is not correlated with the property variables (disease-free survival and duration of cCHRT/RT). Subsequently, PLS is performed on the filtered data to visualize and characterize the biological relationships between the descriptive and property variables. A coefficient overview plot (Figure 1) was used to visualize the influence of the property variables on the descriptive variables analyzed. The OPLS coefficients are a dimensionless measure, usually normalized to the range [−1, 1] to make them comparable when the property variables have different ranges. The coefficient overview plot displays the coefficients for all property variables as vertical bars. The height of the bar shows how strongly the analyzed property variable is associated with a given descriptive variable, and the positive and negative values determine the increase or decrease in the value of a given variable under the influence of the analyzed parameter. The error whiskers represent a 95% confidence interval. The OPLS model was created using internal patient-level cross-validation, and the confidence intervals were derived from jack-knifing uncertainty measures. The same analytical approach was successfully applied in our previous study [58].

To account for the longitudinal nature of the study and prevent the violation of the statistical independence assumptions caused by analyzing weekly consecutive measurements collected from each patient during the treatment, a hierarchical two-stage batch-modeling approach was implemented in SIMCA 18. Individually monitored patients undergoing therapy were treated as separate batches to fully capture the dynamic, non-linear biological response over time.

First, a Batch Evolution Model (BEM) was developed using orthogonal partial least squares (OPLS) regression to capture the multi-dimensional trajectories and fluctuations of the metabolic-nutritional parameters along the treatment time axis.

Second, a batch-level model (BLM) was generated by extracting the unfolded raw data profile from the BEM stage, expanding each subject’s sequential time points into a single-row multivariate profile while fully preserving the chronological structure of the measurements.

Crucially, to prevent data leakage and ensure model robustness, the primary hierarchical BEM/BLM workflow was trained strictly on the training dataset, where a two-class OPLS-DA model was performed on the batch-level dataset to discriminate patients based on disease-free survival (DFS 0 vs. DFS 1). The consistency and validity of the trained training model were rigorously verified using internal cross-validation (Q2), ANOVA of the cross-validated residuals (CV-ANOVA), and permutation testing.

In the subsequent step, an independent external test set validation was conducted. The prediction dataset was processed through a separate batch-level pipeline to generate matching unfolded raw data configurations, which were then projected into the frozen-training BLM space for blind prediction. The final diagnostic and predictive capability of the external test set prediction was evaluated using receiver operating characteristics (ROC)-based area under the curve (AUC) analysis.

Univariate statistical analyzes, including the Mann–Whitney U (MWU) and Chi-squared (χ^2^) tests as well as Kruskal–Wallis ANOVA with Dunn’s correction for multiple comparisons, were performed using Statistica software version 12 (TIBCO Software Inc., Palo Alto, CA, USA). The non-parametric tests were chosen due to non-normal data distribution and increased robustness against outliers. The significance threshold was established at 0.05.

## Figures and Tables

**Table 1:** Characteristics of the study group. Legend: CCI—Charlson comorbidity index, T—primary tumor stage, N—nodal stage, TNM—clinical disease stage, cCHRT—concurrent chemoradiotherapy, iCHT—induction chemotherapy, RT—radiotherapy, CONV—conventional fractionation, CAIR—continuous accelerated irradiation.

	Recovery (*n* = 35)	Recurrence (*n* = 32)	*p*-Value
**Age**			0.58
Median	58 (46–74)	60 (33–73)	
**Localization**			0.47
Larynx	15	11	
Oropharynx	11	7	
Hypopharynx	6	10	
Nasopharynx	3	4	
**Tumor grade**			0.46
1	6	8	
2	24	22	
3	5	2	
**CCI**			0.054
2	7	3	
3	10	12	
4	16	8	
5	1	7	
6	1	1	
9	0	1	
**Stimulants**			0.40
No	2	3	
Cigarettes	11	5	
Alcohol	2	1	
Cigarettes and Alcohol	20	23	
**T**			0.46
0			
1	2	1	
2	7	9	
3	19	12	
4	7	10	
**N**			0.87
0	6	4	
1	6	6	
2	22	20	
3	1	2	
**TNM**			0.54
III	10	6	
IVa	24	24	
IVb	1	2	
**Treatment modality**			0.059
cCHRT	14	19	
iCHT + cCHRT	16	6	
iCHT + RT	5	7	
**RT scheme**			0.62
CONV	30	26	
CAIR	5	7	

**Table 2:** Spearman correlations between the NMR detected lipids, BCAAs and the clinical nutritional parameter in the Recovery and Recurrence groups. Legend: The bold font denotes statistical significance with *p* value < 0.05. ALB—albumin, PreALB—prealbumin. The results take into account the resonance signals of the metabolites at different chemical shifts, e.g., isoleucine at 0.95 and 1.02 ppm.

	Week of RT/cCHRT							
	0	1	2	3	4	5	6	7
Recovery—Spearman correlations R values								
Lipids 0.9 ppm and BMI	**0.36**	0.15	0.18	0.05	−0.03	0.04	−0.03	−0.05
Lipids 0.9 ppm and ALB	0.31	0.04	0.27	0.01	0.06	0.25	0.11	−0.08
Lipids 0.9 ppm and PreALB	**0.71**	0.06	0.06	**0.35**	**0.50**	**0.50**	**0.35**	−0.03
Lipids 1.3 ppm and BMI	0.30	0.18	0.15	0.09	0.14	0.06	0.02	0.06
Lipids 1.3 ppm and ALB	0.28	0.02	0.16	−0.06	−0.12	0.24	0.14	0.00
Lipids 1.3 ppm and PreALB	**0.67**	0.14	0.03	**0.38**	0.33	**0.50**	0.31	0.03
Lipids 5.3 ppm and BMI	**0.43**	0.23	0.13	0.13	0.16	0.09	0.06	0.02
Lipids 5.3 ppm and ALB	**0.40**	0.07	0.19	−0.04	−0.09	0.20	0.13	−0.06
Lipids 5.3 ppm and PreALB	**0.66**	0.15	0.02	**0.35**	**0.50**	**0.46**	0.29	−0.06
Isoleucine 0.95 ppm and BMI	0.21	**0.45**	0.20	0.11	0.00	0.03	−0.02	0.11
Isoleucine 0.95 ppm and ALB	0.03	0.21	0.10	0.29	**0.47**	0.17	**0.38**	0.18
Isoleucine 0.95 ppm and PreALB	0.00	0.00	0.14	0.06	−0.05	0.03	0.06	0.15
Leucine 0.97 ppm and BMI	**0.44**	**0.47**	**0.37**	0.16	0.29	0.03	0.01	0.27
Leucine 0.97 ppm and ALB	0.28	0.29	0.29	0.10	0.29	**0.37**	0.20	0.26
Leucine 0.97 ppm and PreALB	0.21	0.11	0.27	−0.09	0.22	0.11	−0.06	0.27
Leucine 0.98 ppm and BMI	**0.42**	**0.42**	**0.38**	0.19	0.27	0.13	0.24	0.35
Leucine 0.98 ppm and ALB	0.13	**0.42**	0.33	0.21	0.28	0.32	0.28	**0.39**
Leucine 0.98 ppm and PreALB	0.16	0.00	0.29	0.00	0.19	−0.08	0.03	0.35
Valine 1.01 ppm and BMI	0.33	**0.48**	0.34	**0.37**	**0.39**	0.12	0.22	**0.44**
Valine 1.01 ppm and ALB	0.28	**0.50**	0.22	0.20	**0.38**	**0.45**	**0.37**	0.21
Valine 1.01 ppm and PreALB	0.06	0.10	0.29	0.14	0.20	0.12	0.10	0.32
Isoleucine 1.02 ppm and BMI	**0.38**	**0.43**	0.26	0.14	−0.06	−0.06	0.01	0.09
Isoleucine 1.02 ppm and ALB	0.04	0.35	0.19	**0.36**	**0.41**	0.13	0.25	0.24
Isoleucine 1.02 ppm and PreALB	0.09	0.09	0.17	0.11	−0.07	−0.14	0.00	0.20
Valine 1.055 ppm and BMI	0.33	**0.48**	**0.36**	**0.34**	**0.37**	0.12	0.22	**0.41**
Valine 1.055 ppm and ALB	0.32	**0.49**	0.22	0.20	**0.38**	**0.45**	**0.37**	0.27
Valine 1.055 ppm and PreALB	0.07	0.05	0.31	0.11	0.19	0.15	0.09	0.33
Recurrence—Spearman correlations R values								
Lipids 0.9 ppm and BMI	−0.04	−0.12	−0.20	0.05	−0.09	0.07	0.17	−0.27
Lipids 0.9 ppm and ALB	**0.47**	0.30	0.15	**0.42**	0.18	0.16	0.24	0.16
Lipids 0.9 ppm and PreALB	0.33	0.16	0.10	**0.38**	0.06	0.04	0.35	0.16
Lipids 1.3 ppm and BMI	0.08	0.00	−0.05	0.18	−0.06	0.17	0.33	−0.16
Lipids 1.3 ppm and ALB	**0.35**	0.26	0.14	0.28	0.00	0.33	0.18	0.03
Lipids 1.3 ppm and PreALB	0.23	0.17	0.15	0.28	−0.17	0.09	0.27	0.16
Lipids 5.3 ppm and BMI	0.03	−0.07	−0.07	0.08	−0.06	0.18	0.23	−0.16
Lipids 5.3 ppm and ALB	0.33	0.15	0.11	0.27	0.06	0.15	0.09	0.02
Lipids 5.3 ppm and PreALB	0.23	0.05	0.11	0.32	−0.13	0.01	0.21	0.17
Isoleucine 0.95 ppm and BMI	0.33	0.08	0.07	0.00	0.08	0.24	−0.07	0.18
Isoleucine 0.95 ppm and ALB	−0.16	−0.26	−0.23	0.23	0.03	0.04	−0.09	−0.16
Isoleucine 0.95 ppm and PreALB	−0.18	−0.26	0.15	0.09	0.00	0.04	0.15	−0.11
Leucine 0.97 ppm and BMI	0.24	0.03	−0.03	−0.01	−0.05	−0.14	−0.26	−0.05
Leucine 0.97 ppm and ALB	0.07	−0.15	−0.16	0.19	0.05	−0.11	0.00	0.11
Leucine 0.97 ppm and PreALB	0.04	−0.29	−0.02	0.14	0.25	−0.15	0.05	−0.09
Leucine 0.98 ppm and BMI	0.27	0.07	0.19	−0.07	−0.15	−0.08	−0.21	−0.21
Leucine 0.98 ppm and ALB	−0.03	−0.05	0.02	0.03	0.00	0.03	−0.17	0.19
Leucine 0.98 ppm and PreALB	0.06	−0.12	0.29	0.14	0.22	−0.10	−0.08	0.00
Valine 1.01 ppm and BMI	0.23	0.00	−0.07	0.00	−0.04	−0.03	−0.08	0.03
Valine 1.01 ppm and ALB	0.00	−0.01	0.07	**0.37**	0.11	0.13	0.14	0.02
Valine 1.01 ppm and PreALB	−0.07	−0.25	0.18	0.18	0.18	0.08	0.19	0.06
Isoleucine 1.02 ppm and BMI	0.11	0.17	−0.09	−0.01	0.09	0.07	0.01	0.23
Isoleucine 1.02 ppm and ALB	−0.30	−0.36	**−0.39**	0.10	0.03	0.09	−0.01	0.01
Isoleucine 1.02 ppm and PreALB	−0.29	−0.35	−0.19	0.13	0.00	0.00	0.08	−0.09
Valine 1.055 ppm and BMI	0.20	−0.05	−0.12	−0.04	−0.08	−0.05	−0.10	0.00
Valine 1.055 ppm and ALB	−0.02	−0.04	−0.02	0.33	0.08	0.06	0.12	0.04
Valine 1.055 ppm and PreALB	−0.08	−0.31	0.06	0.15	0.15	−0.03	0.22	0.05

**Table 3:** Spearman correlations between the clinical parameters that describe the study group. Legend: The bold font denotes statistically significant correlations (R > 0.3 with *p* < 0.05).

	Age	Tumor Grade	CCI	Stimulants	T	N	TNM	Treatment
Study group								
Age	1							
Tumor grade	−0.04	1						
CCI	**0.85**	−0.08	1					
Stimulants	0.03	−0.05	0.04	1				
T	0	0.02	0.01	0.10	1			
N	−0.04	**0.32**	−0.02	0.05	−0.1	1		
TNM	−0.11	**0.40**	−0.10	0.09	0.17	**0.69**	1	
Treatment	−0.18	0.02	−0.18	−0.20	−0.02	0.20	−0.20	1
Recovery								
Age	1							
Tumor grade	−0.2	1						
CCI	**0.81**	0.2	1					
Stimulants	−0.02	0	−0.16	1				
T	0.05	−0.08	0.11	0.25	1			
N	−0.01	**0.48**	0.03	0.04	0.02	1		
TNM	−0.17	**0.54**	−0.07	0.21	0.14	**0.69**	1	
Treatment	−0.12	−0.23	−0.14	0.12	−0.08	−0.19	−0.24	1
Recurrence								
Age	1							
Tumor grade	0.17	1						
CCI	**0.88**	0.09	1					
Stimulants	0.07	−0.08	0.20	1				
T	−0.06	0.15	−0.07	−0.07	1			
N	−0.1	0.16	−0.11	0.05	−0.23	1		
TNM	−0.06	0.27	−0.18	−0.09	0.19	**0.69**	1	
Treatment	**0.44**	0.21	**0.43**	0.26	0.09	−0.25	−0.20	1

## Data Availability

The datasets generated and/or analyzed during the current study are available from the corresponding author on a reasonable request.

## Supplementary Materials

The following supporting information can be downloaded at https://www.mdpi.com/article/10.3390/ijms27156715/s1.

## Abbreviations

The following abbreviations are used in this manuscript:

χ^2^	Chi squared
3HB	3-hydroxybutyrate
a.u.	arbitrary units
AceAce	acetoacetate
AJCC	American Joint Committee on Cancer
ALB	albumin
AUC	area under curve
BCAAs	branched-chain amino acids
BEM	batch evolution model
BLM	batch-level model
BMI	body mass index
CAIR	continuous accelerated irradiation
cCHRT	concurrent chemoradiotherapy
CCI	Charlson comorbidity index
CONV	conventional fractionation
CPMG	Carr–Purcell–Meiboom–Gill
CRP	C-reactive protein
D2O	deuterium oxide
DFS	disease-free survival
DIFF	diffusion edited
ENT	ear, nose, throat
Glu	glutamate
HCT	hematocrit
HGB	hemoglobin
HGB REC	hemoglobin in reticulocytes
HNSCC	head and neck squamous cell carcinoma
HPV	human papilloma virus
iCHT	induction chemotherapy
JRES	J-resolved
KWA	Kruskal–Wallis ANOVA
LA-HNSCC	locally advanced head and neck squamous cell carcinoma
LYMP	lymphocyte
MONO	monocyte
MPV	mean platelet value
MWU	Mann–Whitney U test
N	nodal stage
NAC	N-acetylcysteine
NAG	N-acetyl-glycoprotein
NEUTR	neutrophil
NMR	nuclear magnetic resonance
NOESY	Nuclear Overhauser Enhancement SpectroscopY
OPLS	orthogonal partial least squares
OPLS-DA	orthogonal partial least squares discriminant analysis
Pcho	phosphocholine
PF	cisplatin and 5-fluorouracil
PLT	platelet
PreALB	prealbumin
RBC	red blood cells
RETIC	reticulocytes
ROC	receiver operating characteristics
RT	radiotherapy
T	primary tumor stage
TNM	clinical disease stage
TPF	docetaxel (Taxotere), cisplatin (Platinol), and 5-fluorouracil
TPR	true positive rate
TSP	trimethylsilylpropionate
UV	unit variance
