# Supplementary material for: Metabolic and Clinical–Nutritional Correlation Patterns in Relation to 3-Year Disease-Free Survival in Locally Advanced Head and Neck Squamous Cell Carcinoma: A Preliminary Analysis
Source: Int J Mol Sci. 2026 Jul 27;27(15):6715. doi: 10.3390/ijms27156715 (PMC13467153; doi:10.3390/ijms27156715)
Supplement: Supplementary file 1 [file ijms-27-06715-s001.zip › ijms-4354601-supplementary.pdf]

Study design

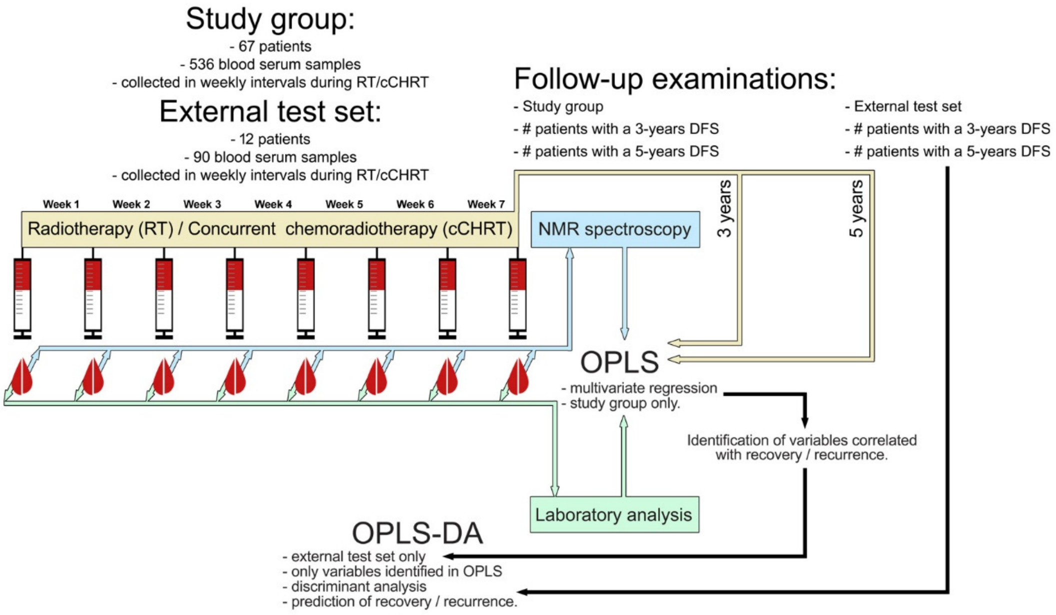

Figure S1. Scheme of the study design.

NMR Measurement protocol and spectra post-processing

The NMR probe tuning and matching, shimming, determination of the transmitter offset value for the water pulse presaturation, and 90° pulse adjustments were always made for each sample. The receiver gain was set to 90.5, and the temperature was set to 310 K for all experiments. Four different 1H NMR spectra were acquired for each serum sample:

- NOESY (Nuclear Overhauser Effect Spectroscopy) – an overview of all types of molecules.
- CPMG (Carr-Purcell-Meiboom-Gill) - information on only low molecular weight metabolites.
- DIFF (diffusion edited) - mainly macromolecular signals.
- Two dimensional (2D) JRES (J-resolved) – visualization of scalar couplings and improved metabolite identification.

Table S1. NMR pulse sequence parameters.

**Pulse program parameters**		**NOESYGPPR1D**	**CPMGPR1D**	**LEDBPGPPR2S1D**	**JRESGPPRQF**
**Time domain**	**TD**	65536	65536	65536	8192
**Spectral width**	**SW** [ppm]	30	20	30	16.62
**Acquisition time**	**AQ** [sec]	2.73	4.09	2.73	0.62
**Relaxation delay**	**D1** [sec]	4	4	4	2
**NOESY mixing time**	**D8** [sec]	0.01	-	-	-
**Delay for gradient recovery**	**D16** [sec]	-	-	0.0002	0.0002
**Delay time between 180° pulses (CPMG) / Diffusion time (DIFF)**	**D20** [sec]	-	0.0003	0.12	-

**Eddy current delay**	**D21** [sec]	-	-	0.005	-
**Dummy scans**	**DS**	4	4	4	16
**Loop for T2 filter**	**L4**	-	126	-	-
**Number of scans**	**NS**	32	64	64	1

The one-dimensional spectra were processed with a line broadening of 0.3 Hz and automatically phase-corrected (in Topspin software version 3.1 from Bruker Biospin), referencing the methyl doublet of alanine at 1.5 ppm and bucketed over the region of 9.0–0.5 ppm with the bucket width set to 0.002 ppm using the AMIX software version 4.0.2 (Bruker Biospin). The spectrum region of water (5.15–4.38 ppm, d = 0.77 ppm) was removed from the analysis in order to prevent variation in each sample. No normalization was applied. This is the standard processing protocol used in our metabolomics lab.

Metabolite Identification and Quantification

The identification of the metabolites was carried out based on the comparisons with the reference compounds library (in Chenomx NMR Suite Professional (Chenomx Inc., Edmonton, AB, Canada)), as well as on the multiplicity and scalar couplings information extracted from the 2D JRES spectra, and using information from the Human Metabolome Database (http://www.hmdb.ca/ accessed on April 18, 2025) and the available literature. The low-molecular-weight metabolites were quantified based on the 1D positive projections of the JRES spectra. The diffusion-edited spectra were used for the quantification of the lipid signals. The integrals were measured in the spectral regions defined individually for each metabolite using the "sum all points in region" method in AMIX software (Bruker Biospin) v.4.0.2.

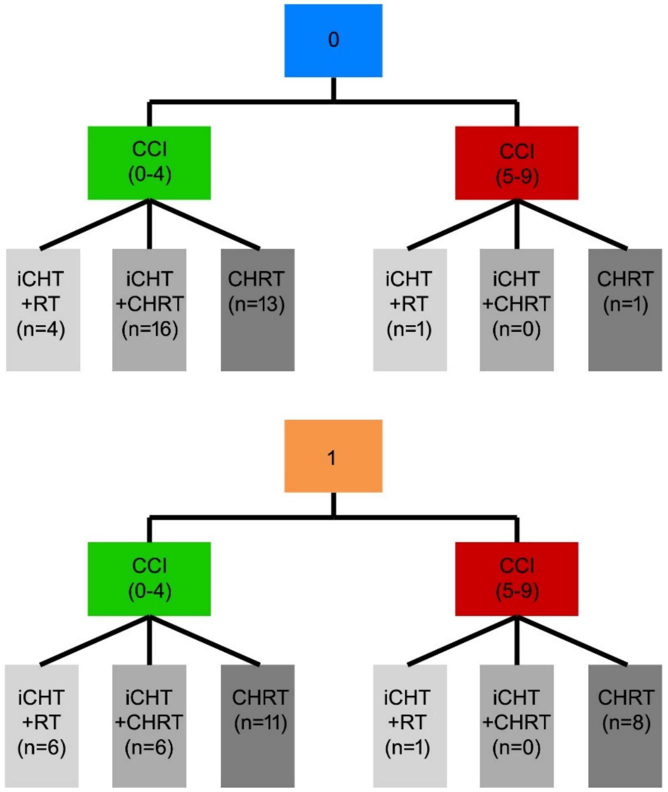

Figure S2. Distribution of the Charlson Comorbidity Index (CCI) values and the treatment modalities within the Recovery and Recurrence classes. This figure, complementary to Table 1, shows that there is more patients with the higher CCI values in the Recurrence class, as well as less patients treated with iCHT+CHRT.

Table S2. OPLS and OPLS-DA models fitting parameters.

**OPLS***					
Predictive				Orthogonal	
Component	R2X	R2	Q2	Component	R2Y
1	0.494	0.0502	0.05	1	0.124
2	0.506	0.0153	0.0142	2	0.101
				3	0.063
				4	0.044
**Batch models**					
**BEM**					
Predictive				Orthogonal	
Component	R2X	R2	Q2	Component	R2X
1	0.209	0.184	0.166	1	0.348
**BLM**					
Predictive					
Component	R2X	R2	Q2		
1	0.261	0.381	0.319		

* OPLS Modeling Approach and Parameter Interpretation

1. Methodological Rationale

To isolate and visualize the specific variables within the large biological dataset that systematically shift in response to the studied parameters, an inverse OPLS modeling strategy was implemented. In this configuration, the traditional matrix assignment was inverted: the time from the start of treatment and the group membership (0-1: Recovery vs. Recurrence) were designated as the predictor block (X), while the blood serum metabolites, laboratory parameters, and clinical markers were treated as the response block (Y).

2. Interpretation of R^2 and Q^2 Metrics

This model was utilized strictly as an exploratory feature-selection and signal-deconvolution tool to extract clean response profiles (regression coefficients), rather than as a forward predictive system. Consequently, the R2 and Q2 metrics exhibit drastically suppressed values. This phenomenon is a direct mathematical consequence of dimensionality asymmetry—specifically, attempting to reconstruct a massive, noisy variable space (Y) using only a few degrees of freedom provided by the limited parameter inputs (X). These low global statistics are expected in inverse PLS/OPLS architectures and do not reflect a lack of underlying biological structure or true covariance.

3. Feature Selection Criterion

Despite the low global fit, the directional alignment of the regression coefficients remains mathematically robust for identifying co-varying trends. To filter out background noise and capture biologically meaningful responses, variables were selected for downstream analysis based on a standardized regression coefficient threshold of $|B| > 0.2$. This multi-step pipeline ensures that only reliable, parameter-driven biological features were progressed to subsequent statistical validations.

For more details please see reference 58.

Table S3. Characteristics of the external test set.

	3-year DFS		5-year DFS	
	Recovery (n=19)	Recurrence (n=17)	Recovery (n=16)	Recurrence (n=20)
**Age**				
Median (min-max)	56 (41-65)	60 (46-73)	56 (41-65)	61 (46-73)
**Localization**				
Larynx	7	9	6	10
Oropharynx	8	5	7	6
Hypopharynx	2	3	2	3
Nasopharynx	2	0	1	1
**Tumor grade**				
1	6	7	5	8
2	6	10	4	12
3	6	0	6	0
No id	1	0	1	0
**CCI**				
2	2	1	2	1
3	9	3	8	4
4	5	6	3	8
5	0	5	0	5
6	2	1	2	1
7	0	1	0	1
**Stimulants**				
NO	6	2	6	2
Cigarettes	2	2	2	2
Alcohol	1	1	1	1
Cigarettes and Alcohol	10	12	7	15
**T**				
1	2	0	1	1
2	4	2	4	2
3	10	11	9	12
4	3	4	2	5
**N**				
0	4	10	3	11
1	2	2	2	2
2	8	4	8	4
3	5	1	3	3
**TNM**				
III	5	8	5	8
IVa	9	8	8	9
IVb	5	1	3	3
**Treatment modality**				

RT	3	10	3	10
cCHRT	3	4	3	4
iCHT+cCHRT	11	2	9	4
iCHT+RT	2	1	1	2
**RT scheme**				
CONV	15	7	12	10
SIB	1	0	1	0
CAIR	3	10	3	10
**HPV status**				
Unknown	8	2	5	5
HPV-	3	10	3	10
HPV+	8	5	8	5

Legend: CCI – Charlson Comorbidity index, T – primary tumor stage, N – nodal stage, TNM – clinical disease stage, cCHRT – concurrent chemoradiotherapy, iCHT – induction chemotherapy, RT – radiotherapy, CONV – conventional fractionation.

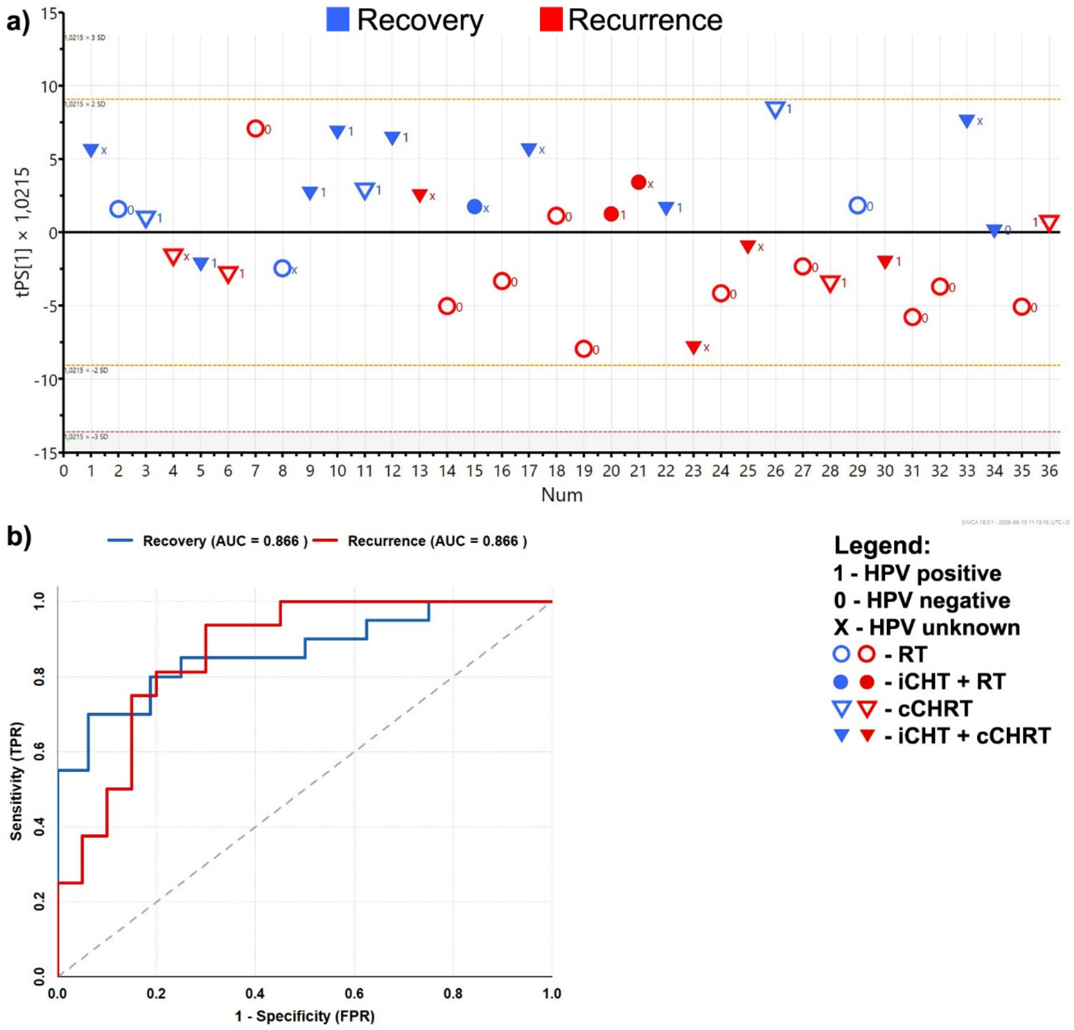

Figure S3. The results of the 5-year DFS prediction for the external test set. Data from 36 patients were used for prediction. The plot of the predicted scores (tPS) (a) shows the results obtained by the OPLS-DA Batch Level model constructed for the study group. The receiver operating curve (b) shows a high predictive capacity of the model with an area under the true positive rate (TPR) curve of 0.866. The significance of the observed classification was evaluated with Fisher's probability test with p-value = 0.0002.
